# Amphibian infection tolerance to chytridiomycosis

**DOI:** 10.1098/rstb.2022.0133

**Published:** 2023-07-31

**Authors:** Laura F. Grogan, Madelyn J. Mangan, Hamish I. McCallum

**Affiliations:** Centre for Planetary Health and Food Security, and School of Environment and Science, Griffith University, Gold Coast Campus, Parklands Drive, Southport, Queensland 4222, Australia

**Keywords:** disease, frogs, host, defence, pathogen, animal

## Abstract

Animal defences against infection involve two distinct but complementary mechanisms: tolerance and resistance. Tolerance measures the animal's ability to limit detrimental effects from a given infection, whereas resistance is the ability to limit the intensity of that infection. Tolerance is a valuable defence for highly prevalent, persistent or endemic infections where mitigation strategies based on traditional resistance mechanisms are less effective or evolutionarily stable. Selective breeding of amphibians for enhanced tolerance to *Batrachochytrium* spp*.* has been suggested as a strategy for mitigating the impacts of the fungal disease, chytridiomycosis. Here, we define infection tolerance and resistance in the context of chytridiomycosis, present evidence for variation in tolerance to chytridiomycosis, and explore epidemiological, ecological and evolutionary implications of tolerance to chytridiomycosis. We found that exposure risk and environmental moderation of infection burdens are major confounders of resistance and tolerance, chytridiomycosis is primarily characterized by variation in constitutive rather than adaptive resistance, tolerance is epidemiologically important in driving pathogen spread and maintenance, heterogeneity of tolerance leads to ecological trade-offs, and natural selection for resistance and tolerance is likely to be dilute. Improving our understanding of infection tolerance broadens our capacity for mitigating the ongoing impacts of emerging infectious diseases such as chytridiomycosis.

This article is part of the theme issue ‘Amphibian immunity: stress, disease and ecoimmunology’.

## 1. Tolerance and resistance are complementary host defences against infection

Animal defences against infection involve two distinct but complementary mechanisms: tolerance and resistance. Tolerance measures an animal's ability to limit detrimental effects from a given infection, whereas resistance is the ability to limit the intensity of that infection (box 1; for a precise statistical definition that is suitable for many disease contexts, please see [3]). The concept of infection tolerance has revolutionized our understanding of animal host defences against pathogens since it was first applied from plant biology over a decade ago [8]. Tolerance has been of particular interest lately for diseases that are highly prevalent, endemic, chronic, persistent, and where mitigation strategies based on traditional resistance mechanisms (including antimicrobials, vaccines and selection for resistance) are less effective or evolutionarily stable (e.g. malaria, tuberculosis and infections with protozoa, fungi and helminths; [14–17]). The devastating amphibian skin disease, chytridiomycosis, caused by fungal pathogens *Batrachochytrium dendrobatidis* and *B. salamandrivorans* (hereafter Bd/Bsal) falls under this umbrella of traits. However, despite an early call to action by Venesky *et al*. [18], and widespread acknowledgement of the general importance of infection tolerance across scales and disciplines, remarkably little attention has been paid to this component of host defence in chytridiomycosis systems thus far. Here, we argue for the importance of understanding infection tolerance as it relates to chytridiomycosis, from both basic theoretical and applied conservation management perspectives.

Box 1. **Glossary.**

**Avoidance** is an active behavioural strategy to reduce the risk of exposure to infectious agents first requiring detection (olfactory/gustatory/visual/auditory) of risk of pathogen exposure prior to infection [1]. May be innate or learned.

**Carrier** is an infected host capable of transmitting infection while remaining free of clinical signs [2].

**Figure 1.**
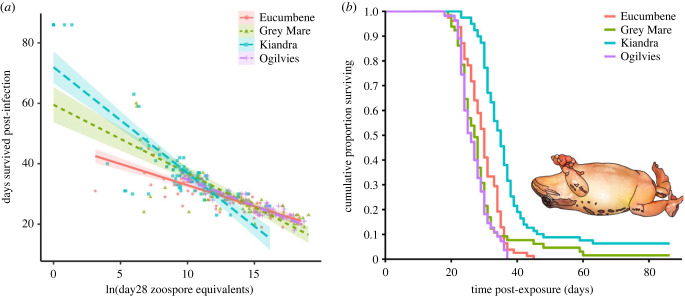
Example of evidence for variation in tolerance to chytridiomycosis. (*a*) Fitness (measured as days survived post-exposure) against pathogen burden (measured as the natural logarithm of the zoospore equivalents [zse] load measured via skin swab qPCR at day 28 post-exposure for all individuals, chosen to avoid confounding from mortality) regression lines separated by population (Eucumbene, Grey Mare, Kiandra and Ogilvies) for the alpine tree frog (*Litoria verreauxii alpina*). There was a significant interaction (varying regression slopes) between zse and population via ANCOVA. (*b*) Survival curves for the same animals from (*a*), separated by population. Note the long subclinical period prior to mortality (clinical signs only manifest in the last 12–48 h prior to mortality). Importantly, the infection loads at time of death ranged up to 8 million zse in this species. Data sourced from Grogan *et al.* [19].

**General vigour** is baseline host fitness in the absence of infection/exposure (box figure 1*a*; [6])

**Reservoir** is a population or environment that can permanently maintain a pathogen and can transmit the pathogen to a target population [7].

**Resistance** is the ability of the host to prevent infection when exposed (qualitative resistance) or limit pathogen burden (quantitative resistance) [6]. Resistance protects the host at the cost of the pathogen. It is commonly measured as the inverse of infection intensity given a specified infection dose (lower intensity implies higher resistance, box figure 1*a* [8]), or as the inverse of growth rate of pathogen burden on a host given an initial infection burden (lower growth rates correspond with higher resistance, box figure 1*b* [9,10]). A statistical definition of resistance is provided by Råberg *et al*. [3], but importantly is not applicable to all infectious disease contexts due to system-specific sources of confounding.

**Super spreader** is a host individual, population or species that increases the risk of infection for other hosts [11].

**Susceptibility** is variably used in common parlance to imply the inverse of both resistance and tolerance, but we propose that its use be made more explicit. Here, we define *susceptibility to infection* as the relative ability of the host to become infected (inverse measure of qualitative resistance), while *susceptibility to disease and subsequent mortality* is the relative ability of the host to sustain clinical signs (disease) and/or mortality resulting from the infection (inverse measure of tolerance).

**Tolerance** is the ability to limit the detrimental effects of infection from a given pathogen burden [8], not to be confused with immunological tolerance (non-responsiveness to self-antigens; [1]). Tolerance protects the host without harming the pathogen [3]. It is commonly measured as the rate of change in fitness as pathogen burden increases (individual; [12]) or the slope of host fitness against peak pathogen burden (group), where the steeper the slope, the lower the tolerance (box figure 1*a*; [9]). Råberg *et al*. [3] present an explicit statistical definition for comparing tolerance between host types, but importantly, appropriate measures for tolerance can be highly context specific due to system-specific sources of confounding and this field is necessarily still evolving.

**Virulence** is the ability of a pathogen to cause disease in a given host and comprises both host and pathogen factors, including host genes affecting (i) infection intensity (resistance) and (ii) damage per pathogen (tolerance), and pathogen genes affecting (i) infection intensity (exploitation) and (ii) damage per pathogen (per pathogen pathogenicity) [13].

**Box Figure 1.**
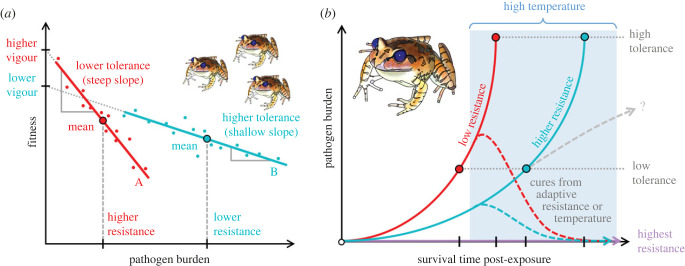
Schematics depicting approaches to measure tolerance and resistance at group and individual scales. (*a*) At the group scale, resistance is classically measured as the inverse of mean peak infection intensity (unless that peak occurs at time of death), tolerance is the slope of a regression of host fitness against peak infection intensity, and general vigour is fitness in the absence of infection. Host types (A, red; B, cyan, etc.) can be different species, populations, genotypes, life stages, sexes, etc. Adapted from Råberg *et al.* [3]. (*b*) At the scale of an individual chytridiomycosis infection, resistance manifests as the rate of growth of pathogen burden post-exposure, ranging from complete (qualitative) resistance (violet), intermediate constitutive resistance (cyan) through mechanisms such as effective antimicrobial peptides and symbiotic microbiota [4], and low resistance (red) where exponential pathogen growth approaches the intrinsic growth rate of the pathogen in culture. The dashed grey line implies changing (increasing) resistance through time, fundamentally promoting carrier states (potential causes of which are discussed in the text). Animals with low tolerance will die at low pathogen burdens relative to animals with high tolerance, with corresponding survival times post-exposure. Infection cures associated with efficacious adaptive resistance (dashed red and cyan lines) are uncommon in highly susceptible species; however, similar but largely non-heritable resolutions of infection can be caused by exposure to high environmental temperatures (blue background shading; [5]). Importantly, animals must survive long enough in the field for such cures to occur, and hence both resistance and tolerance are important in determining their occurrence.

### (a) Tolerance mechanisms counter pathogenesis

In practical disease settings, tolerance is typically interpreted as comprising mechanisms that counter the pathological outcome of infection (i.e. morbidity/mortality) or promote production/yield (in agricultural settings) as opposed to those that promote Darwinian fitness (lifetime reproductive success; [3]). Infection pathology manifests as damage to host parenchymal tissues and metabolic dysfunction. Tolerance mechanisms operate at the cellular, tissue/organ and systemic scales to minimize detrimental effects of infection, while sustaining functional outputs. Post-metamorphic amphibians infected with Bd/Bsal commonly exhibit relatively long subclinical periods (e.g. three weeks; figure 1*b*; [19]), implying that the host can tolerate infection up to a certain pathogen load, time post-exposure or some combination of factors. However, when infection pathology starts to systemically compromise host homeostasis, the intrinsic capacity of tolerance mechanisms has been exceeded and clinical signs of disease emerge [1,12].

Pathology derives from mechanisms relating (i) directly or indirectly to the infecting pathogen itself, and/or (ii) to dysregulated host defence mechanisms [1,12]. In the case of chytridiomycosis, Bd/Bsal cause *direct damage* by invading epidermal tissues and intracellularly hijacking epithelial cells [20], compromising the functional integrity of the skin and leading to systemic electrolyte imbalances (Bd; [21]) or lethal septicaemia (Bsal; [22,23]). They also cause *indirect damage* by releasing toxic and locally immunosuppressive metabolites that cause lymphocyte apoptosis [24]. Via a highly resource-consumptive, largely ineffective and massively dysregulated immune response, this latter virulence factor is thought to contribute to the second source of pathology: *immunopathology* [25–28]. Therefore, tolerance to chytridiomycosis can involve mechanisms that (i) mitigate direct or indirect damage and functional compromise, and/or (ii) reduce or prevent immunopathology. Corresponding to the first, such mechanisms likely include stress responses (removal of oxygen free radicals, promoting anaerobic metabolism and cytoprotection), damage responses (inhibiting lipid peroxidation, limiting DNA damage, autophagy to remove damaged molecules), and tissue regeneration and repair. Second, mechanisms combatting immunopathology likely include immune regulatory factors, anti-toxin immunity (e.g. antibodies against secreted virulence factors) and improved efficacy of antigen-specific adaptive immunity (limiting collateral damage) [12]. While not yet definitively demonstrated for amphibians, the genes and proteins involved in these putative tolerance mechanisms are common across vertebrates and provide promising leads for future studies.

### (b) Variation in resistance and tolerance features unequivocally in chytridiomycosis systems

Relatively unconfounded evidence of variation in resistance (i.e. variation in susceptibility to infection and resultant pathogen loads) between species, populations, individuals and treatments has been repeatedly demonstrated throughout the experimental chytridiomycosis literature [19,29,30]. This is likely due to the ease of non-invasively and longitudinally sampling Bd/Bsal loads from the same individuals over time via skin swabbing techniques and quantitative polymerase chain reaction (qPCR) diagnostics (note: load measures taken at time of death are typically not appropriate for measures of resistance as they are confounded with tolerance). By contrast, although some clear examples of variation in tolerance exist (figure 1*a*), most mentions of the terms ‘tolerance’ and ‘tolerant’ within the chytridiomycosis literature to date (e.g. from 80 results for Title-Abstract-Keywords search ‘*Batrachochytrium* AND toleran*’ among 1808 results for search ‘*Batrachochytrium*’ via Scopus [Elsevier], 21 July 2022) have (i) been non-explicit in relation to currently preferred definitions (box 1; [3]), (ii) lacked supporting evidence, (iii) involved binary classification (tolerant versus non-tolerant) and been applied at either the species level or to individual survival data, and/or (iv) been confounded with resistance or exposure risk where scalar quantitative data exist.

Despite these challenges, there are at least two clear generic examples that illustrate the presence and importance of variation in infection tolerance in chytridiomycosis systems more generally. First, in most anuran species, tadpoles can become infected with Bd with high infection prevalence and develop high infection intensities, yet rarely die pre-metamorphic climax as a direct result of infection (note: larval results from [31] were not constrained to mortality occurring pre-metamorphic climax; [32–34]). This is in stark opposition to the devastating mortality rates in post-metamorphic amphibians, where up to 100% of individuals might die from infection during experimental trials (figure 1*b*; [19,35]). Second, Bd/Bsal infections in post-metamorphic amphibians often involve long subclinical periods (e.g. several weeks) during which time animals experience (tolerate) a wide range of infection intensities that elicit neither clinical signs of disease, nor mortality in the immediate term (figure 1*b*; [19,36]). While survival time is confounded with resistance (animals survive longer if their pathogen growth rate is lower), here we are using the phrase ‘long subclinical period’ to refer explicitly to the temporal implications of surviving for a period of time with high pathogen loads (high tolerance). We will elaborate on these two examples in the following discussion of the epidemiological, ecological and evolutionary implications of tolerance.

## 2. Why do infection tolerance and resistance matter in chytridiomycosis systems?

Chytridiomycosis systems provide several unique and fascinating challenges to our expanding understanding of the concept of infection tolerance, while concurrently providing strong applied conservation justifications.

### (a) Environmentally mediated factors are major confounders of resistance and tolerance in the field

**Figure 2.**
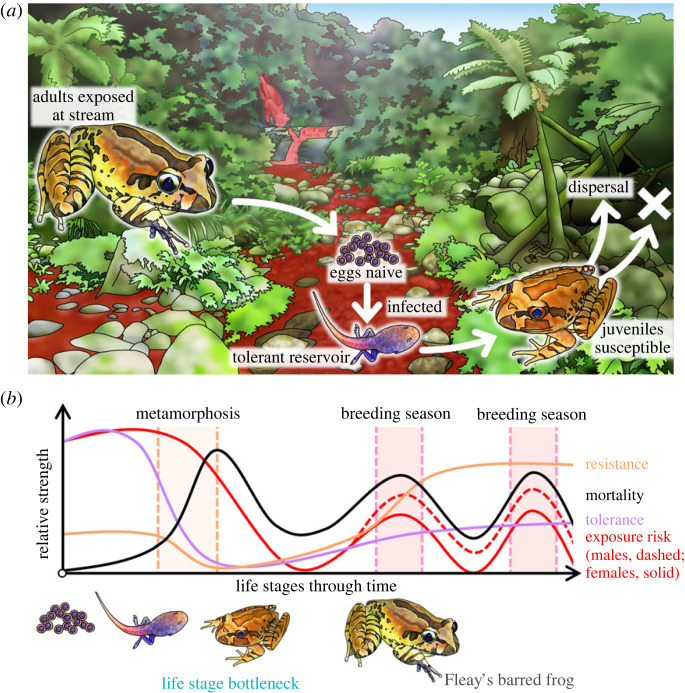
Example of epidemiological and ecological implications of infection tolerance to chytridiomycosis. (*a*) In the rainforests of eastern Australia, the tadpoles of the Fleay's barred frog (*Mixophyes fleayi*) act as a tolerant reservoir, maintaining an environmental zoospore pool year-round causing high-exposure risk within the stream (represented in red). As these tadpoles metamorphose, many are expected to die from chytridiomycosis (unpublished data; [37]), while those that survive disperse from the stream, potentially spreading infection to naïve catchments due to their long subclinical period (unpublished data). Adults are exposed to infection as they come to the stream to breed, with males maintaining territories along the stream year-round [38]. Eggs are initially naïve to infection, but the tadpoles soon become infected after hatching. (*b*) Hypothetical depiction of changes in life-history-related exposure risk independent of seasonal temperature changes (red lines, separated for adults into females [solid line] and males [dashed line]), resistance (orange line), tolerance (violet line) and mortality probability (black line) throughout the course of an individual amphibian's lifespan. Ecologically, the high-exposure risk at the stream combined with very low tolerance and resistance at metamorphosis results in high mortality of the metamorphosing amphibians, leading to a likely life stage bottleneck (unpublished data; [37]).

Variable exposure risk is a key determinant of infection dynamics and can be a major confounder of measures of both resistance and tolerance. At its simplest and in the case of a controlled experiment, exposure risk is a function of the infective dose to which a host is exposed [8]. In practice, exposure risk is influenced by exposure duration and frequency, and can vary with species-specific habitat use, and life stage-, age- and sex-specific behaviours within species (e.g. aquatic larvae, dispersal stages and male maintenance of territories; figure 2*a*). For most amphibians, exposure risk is proportional to the amount of time spent associating with one of two main sources of transmission: (i) common environmental pool (usually aquatic) maintained by reservoir hosts (Bd; [39,40]) or a self-perpetuating environmental reservoir (Bsal; [41]), or (ii) direct contact between conspecifics (e.g. during breeding). In association with these transmission sources, exposure risk is further mediated by (i) the proximity to and relative density of infectious zoospores in the environmental pool [23,42,43], or (ii) the relative density and shedding rate of conspecifics that are in direct contact [44]. Clinically infected amphibians are anecdotally reported to give off a recognizable smell (likely associated with the metabolite putrescine; [28]), and there is currently some evidence to suggest that amphibians may be able to detect and actively avoid [1,45] infected conspecifics or other sources of infection thereby reducing exposure risk [46]. Regardless of potential avoidance behaviours, however, variable exposure risk is thought to be a confounder of resistance and tolerance measures. Unfortunately, variation in exposure risk, particularly for cryptic species with an environmental route of transmission, cannot typically be identified from field data (even on longitudinally recaptured animals). Thus, without very detailed knowledge of behaviour and habitat use of Amphibia in the same community, assessing the extent to which the different species have similar exposure risk is challenging. It is therefore very difficult to evaluate species or individuals as more or less resistant or tolerant from field data in the absence of controlled laboratory experiments.

Variable environmental conditions are unusually important mediators of infection probability and maintenance within chytridiomycosis systems due to Bd/Bsal being temperature- and moisture-dependent pathogens that infect the skin of ectothermic hosts (Bd and Bsal optimal thermal ranges are 17–25 and 10–15°C, respectively [47–49]). Environmental conditions vary with microhabitat use, with basking, the use of aquatic versus terrestrial retreat sites, time spent in different locations and stochastic fluctuations of environments both daily and seasonally. As demonstrated via laboratory experiments, elevated temperatures can completely resolve infections [5,50]. However, in the absence of an effective adaptive immune response, it is unlikely that such cures involve protective immunological memory, hence most animals experiencing thermal cures should return to the susceptible state [4]. We only consider behavioural differences that relate to environmental mediation of infection to be features of intrinsic resistance and tolerance where the behaviour is demonstrated to be an active response to infection. Despite early interest in behavioural fever, results from laboratory experiments suggest there is currently little evidence that subclinically infected animals actively alter their behaviour to effect temperature-mediated cures, and any observed associations from field data are likely instead related to individual thermal preferences and non-census sampling [51,52]. By contrast, there is preliminary evidence to suggest that some species (e.g. alpine newts) can actively alter their behaviour to reduce infection by preferencing dryer environments [53]. However, for the most part, a host's variable experience of environmental conditions is thought to confound measures of intrinsic resistance and tolerance. Interestingly, the probability of an infected animal surviving long enough to experience an environmentally mediated cure could, however, be related to their degree of intrinsic resistance and tolerance (box figure 1*b*). Aside from sampling biases contributing to noise in longitudinally recorded pathogen loads, variation in environmental conditions is likely a major source of fluctuations commonly observed in field studies [5]. It must also be noted that variation in pathogen strain can also confound measures of resistance and tolerance.

### (b) Variation in constitutive (rather than adaptive) resistance is a major feature of chytridiomycosis systems

After accounting for variation in exposure risk and environmental conditions, variation in constitutive (always present) resistance (box figure 1*b* cyan and violet lines) is the major determinant of species' susceptibility to chytridiomyosis [44,54]. This has been demonstrated by studies involving functional depletion of constitutive immune components such as antimicrobial peptides and skin microbiota [4,55–57]. Importantly, although potentially heritable and modulated by the presence of microbiota [58], components of constitutive resistance typically cannot be adequately induced or acquired during the course of infection. That is, their protective effect does not scale with infection burden and hence animals typically cannot self-cure via this mechanism except through environmental mediation (box figure 1*b* dotted cyan line; [27]). Nor does the presence of constitutive resistance result in immunological memory post-infection (i.e. infections have no lasting protective effect on subsequent exposures, although consider innate immune memory; [59,60]). Thus, infections can become persistent often with increasing pathogen burden trajectories and can result in mortality if pathogen optimal environmental conditions are maintained.

In some scenarios, amphibians might maintain relatively stable infection loads through time and hence act as carriers longer term (box figure 1*b* dashed grey line; [2]). Such load equilibria could occur where pathogen growth is intrinsically logistic, limited by resource availability (as occurs in culture media; [61]) and tissue tropisms (e.g. limited to the mouthparts of tadpoles). Alternatively, equilibria could occur through some combination of factors that over time reduce Bd growth while also reducing the immune response. Factors that reduce or limit Bd growth could include (i) temperatures that are suboptimal for Bd growth, (ii) a pathogen that exhibits low activity/dormant resistant spore (Bsal; [62]) or latent states, (iii) low replicability of less virulent strains of the pathogen [63], or (iv) removal by skin sloughing. Factors that could limit the immune response could include (i) some form of constitutive or induced immunity such as the protective effect of skin sloughing [64] in the absence of a fully protective adaptive immune response, (ii) a pathogen that employs immunoevasive or immunosuppressive strategies [24,65,66] or (iii) a host that exhibits some form of immunological tolerance (low activation state).

There are currently very few examples of amphibian species that demonstrate an efficacious and protective acquired immune response to chytridiomycosis (sufficient to completely resolve or prevent infection either through induced innate or adaptive immune priming and memory; box figure 1*b* dashed lines; [4,29,67]). There are several potential reasons for this, not limited to Bd/Bsal being intracellular eukaryotic pathogens that secrete locally immunosuppressive metabolites including methylthioadenosine, kynurenine and spermidine [24,65,66]. We expect that most examples of the resolution of infection in the field are thus associated with environmental mediation (e.g. elevated temperatures) rather than the development of adaptive resistance.

### (c) Tolerant hosts disproportionately drive epidemiological dynamics towards global pathogen endemism

Even in demographically open host–pathogen systems, it is not unexpected for either highly fatal or immunizing infections to die out epidemiologically (at least at the local scale), through rapid mortality or recovery of infected hosts (causing the effective reproductive number of the infection to drop below one, *R*_e_ < 1; [68,69]). However, despite being a highly fatal infection in many amphibian species (figure 1*b*; [35,70]), three key features of chytridiomycosis are thought to be responsible for promoting the wide geographical persistence of the Bd global panzootic lineage (BdGPL) and subsequent shift towards pathogen endemism [71–73]: (i) Bd has an indirect (environmental) route of transmission, with the potential to survive weeks to months in the environment [43,61,74]; (ii) Bd can be maintained environmentally via tolerant host life stages and through the long subclinical period of infected hosts; and (iii) Bd is a generalist multi-host pathogen. Below, we describe these features of chytridiomycosis systems and illustrate the important role of tolerance in driving this spread and maintenance of Bd in particular.

The presence of an environmental zoospore pool (typically a stream, pond or lake/dam) is an important route of transmission for Bd/Bsal, and via modelling approaches has been demonstrated to substantially increase pathogen invasiveness [44]. Although ancestrally saprotrophic, of the pathogenic chytrids that infect vertebrate hosts, only some isolates of Bsal have apparently retained this ability to replicate environmentally [41]. While Bd can survive for months in sterile moist river sand [74], there is little evidence for the presence of a competent abiotic environmental reservoir outside sterile culture conditions [75,76]. Thus, the environmental zoospore pool must be maintained by infected hosts. Those hosts with the greatest capacity to shed large numbers of zoospores include animals with relatively low resistance and high tolerance, such as tolerant larvae and infected post-metamorphic amphibians during the long subclinical period [32,34]. By definition, tolerance does not detrimentally affect pathogen growth rates [3]. However, by improving the fitness (i.e. extending survival via mortality tolerance; [77]) of infected hosts, increasing tolerance in an amphibian community generally is expected to have a net positive effect on the force of infection in that same community, increasing (i) pathogen transmission, (ii) infection prevalence and (iii) infection intensities, in the immediate term [6,78].

Tolerant infected tadpoles are a particularly important source of environmental zoospores due to their aquatic lifestyle and water's suitability as a medium for pathogen dispersal independent of hosts (figure 2*a*). This may be especially true of lotic systems, where infected amphibians inhabiting headwater streams can act as a source of infection to downstream species via water flow. However, based on the results of host density transmission experiments, it is likely that the distribution of infectious zoospores in the aquatic environment is concentrated around highly shedding hosts and thus declines with dilution downstream [43,79]. Terrestrial post-metamorphic amphibians also make regular use of these aquatic systems for male advertisement calling, maintenance of territories and breeding, thus generating habitat niche overlap and potentiating infection transmission between life stages.

Depending on the amphibian species, some tadpoles have very long developmental periods (e.g. *Mixophyes fleayi* tadpoles can take 1–2 years to metamorphose and have high Bd infection prevalence and intensity in the field, unpublished data; figure 2*a*; [80]). These long-lived tadpoles with high infection prevalence that do not die from infection can act as a continuous biotic reservoir, maintaining the infection source year-round [32,34,81]. Any sublethal developmental effects on tadpoles due to loss/malformation of their mouthparts could also result in greater temporal homogenization of the environmental pool [82,83]. In other systems, there might be more distinct seasonal fluctuations in the infectious capacity of the environmental pool, mediated by (i) peaks in the number and size of occupying larvae and adults, and/or (ii) changes in seasonal thermal suitability for pathogen replication and survival [42,47]. Such fluctuations could generate pulses of new infections or trade-offs in infection dynamics. For example, while winter temperatures are optimal for pathogen growth in the rainforest streams of eastern Australia, *M. fleayi* adults spend more time at the stream during the summer breeding season, effectively moderating high exposure risk against suitable environmental conditions (figure 2*a*; [38]).

Regardless of whether they go on to die from infection, experience resolution, or remain infected as carriers, post-metamorphic amphibians typically demonstrate a long subclinical period (e.g. weeks). If they succumb to chytridiomycosis, clinical signs will usually only emerge immediately prior to death (often in the last 24–48 h; [84]). Thus, subclinically infected animals continue to behave relatively normally (e.g. breeding successfully) for weeks post-exposure and thus may be able to spread large amounts of pathogen (figure 1*b*; [19]). In some cases, their interactions with conspecifics may even be enhanced during this period [85]. Epidemiologically, infected dispersing classes (e.g. juveniles/subadults and adult females) that migrate between breeding and non-breeding habitats are candidates for super spreading, capable of introducing the pathogens into previously naïve systems (figure 2*a*; [11]). By contrast, acute cases that are pre-morbid, with the highest tolerance and lowest resistance (i.e. the potential to reach the highest infection loads) likely function as super shedders.

The presence of an environmental zoospore pool that is frequented by sympatric species also dramatically increases the probability of cross-species transmission, by acting as a common infection source [86]. Both Bd and Bsal have demonstrated exceptionally broad host ranges across the Lissamphibia, including the propensity to cause declines and extinctions across taxonomic groups [87,88]. Epidemiologically, increased species richness and cross-species transmission via a common environmental pool has the potential to either amplify or dilute epidemic outbreaks, depending on characteristics of the species with greatest epidemic potential, as demonstrated by Espira *et al.* [89]. However, Wilber *et al*. [90] demonstrated through modelling that single host species were primarily responsible for promoting pathogen persistence in the majority of metacommunities they examined. This effect is likely associated with a number of factors including life-history characteristics (e.g. multi-year tadpole stages), autecology (e.g. differences in habitat use between species), relative abundance/density, and the relative degree of tolerance and/or resistance of the species as a whole and subpopulations therein [44,90]. Nevertheless, the broad host range of Bd/Bsal expands the potential geographical distribution and niche breadth occupied by the pathogens, together with promoting a more homogeneous temporal presence as the sum of the individual species' autecology.

### (d) Tolerance heterogeneity within and between species leads to ecological trade-offs for population persistence

As a recently globally emerged infectious disease, chytridiomycosis, specifically caused by BdGPL, is expected to elicit heterogeneity of host tolerance in most systems. Such heterogeneity within and between species can lead to ecological trade-offs and potential shifts in resulting abundances and distributions in the immediate term. Although expected to be rare, high homogeneity of tolerance should promote population persistence and community stability through the mechanism of increased survival [3,77]. Where high heterogeneity occurs, there can be a range of system- and context-specific components driving outcomes, relating to aspects of species life-history and community structure. As already described, cross-species transmission will occur where communities interact with a common environmental zoospore pool [86]. However, the extent of detriment from chytridiomycosis (i.e. mortality and suppression of population growth rates) should be related most strongly to the respective species’ intrinsic levels of resistance and tolerance, assuming equivalent exposure and similar autecology [44].

**Figure 3.**
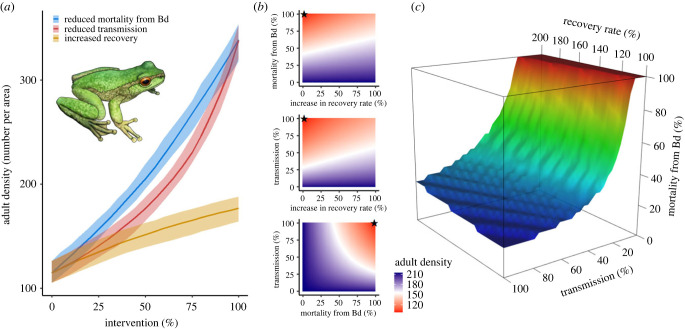
Example of leveraging components of resistance and tolerance in a species under pathogen pressure. The critically endangered spotted tree frog (*Litoria spenceri*) continues to decline throughout its range in southeast Australia, in large part due to pathogen-mediated competition from syntopic reservoir hosts such as the stony creek frog (*L. lesueurii*) [93]*.* Here, we demonstrate hypothetical results of leveraging tolerance (host mortality rate due to Bd) and multiple facets of resistance (transmission rate, recovery rate) in improving conservation outcomes of a species under strong pathogen pressure from the amphibian community. In all panels, various two-species, stage-structured, SI compartmental models were run from quasi-equilibrium to characterize 20-year outcomes for *L. spenceri* adults sharing a zoospore pool with a population of *L. lesueurii*. (*a*) Median (±95% bootstrap confidence interval) of *L. spenceri* adult density after 20 years of implementing varying interventions across 1000 equilibrated parameter sets. At intermediate values, reduced mortality due to Bd (tolerance) more efficiently rescues *L. spenceri* populations than equivalent reductions in transmission rate. Doubling the recovery rate from infection offers only marginal benefits without concomitant changes in transmission or reductions in mortality. (*b*) Heatmap comparisons of 20-year *L. spenceri* outcomes in two dimensions, increasing recovery rate by 0–100% of its baseline value while decreasing mortality due to Bd and transmission rates from 100% to 0% of their baselines. Black stars indicate the original model (without parameter modifications, from field-realized parameter estimates) in each panel. Combined reductions in Bd-associated mortality and transmission offer the strongest population outcomes for *L. spenceri*. (*c*) The three-dimensional relationship across varying levels of Bd-associated mortality, transmission and recovery rates, where the barrier density plane represents the median 20-year adult density of *L. spenceri* across all scenarios. High-density outcomes of *L. spenceri* fall below the plane, while low densities are represented above the plane.

Via pathogen-mediated apparent competition, more resistant and tolerant species are expected to be ecologically favoured overall (limited primarily by their intrinsic population demographic rates) facilitating the declines and potentially extinction of less tolerant species that lack sufficient resistance [91]. These resistant and tolerant species might come to dominate a community, particularly if less resistant/tolerant species experience declines leading to the opening of niche space [92]. The relative importance of resistance versus tolerance for promoting survival and population persistence is expected to be pathogen-specific [1,3] and is not yet clear for chytridiomycosis systems, but should depend not only on the intrinsic dynamic range of the effect itself, but also the frequency of its expression across the population. The magnitude and temporal persistence of infection pressure from the amphibian community can also influence the relative advantage conferred by resistance and tolerance. For example, under heavy force of infection from syntopic reservoir species, small gains in resistance may provide less benefit towards survival than equivalent gains in tolerance (figure 3). Alternatively, when infection pressure is low or seasonally intermittent, small gains in resistance may more efficiently expedite the rate of environmentally mediated cures by limiting pathogen burden. However, regardless of these contextual trade-offs, any development of increased resistance and/or tolerance in a target species (whether overall or limited to a subpopulation) should generally be favourable to that species.

**Figure 4.**
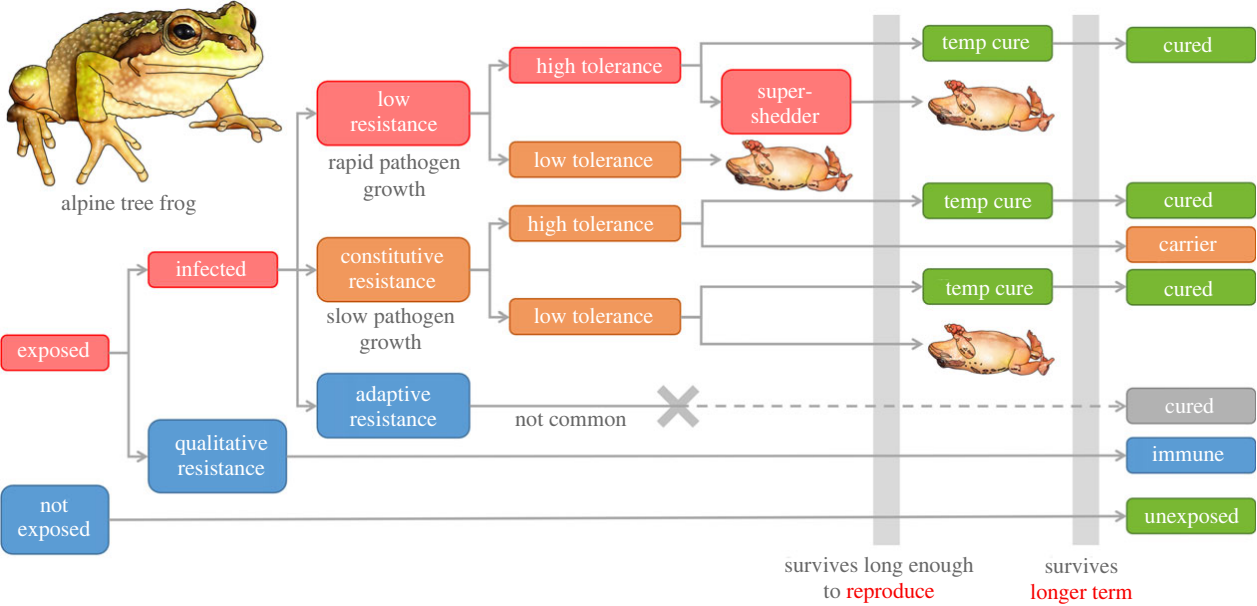
Example classification tree demonstrating the evolutionary implications of the long subclinical period. The alpine tree frog (*Litoria verreauxii alpina*) provides an important case study when considering the potential evolutionary implications of the long subclinical period of chytridiomycosis. In this system, adults return to waterbodies to breed, rapidly become infected via the environmental zoospore pool and generally breed successfully, before the majority of them go on to perish [94]. Here, we have an example of where selection via differential reproductive success fails to occur in the breeding cohort due to the long subclinical period. In this system, the age structure has been dramatically truncated, with the majority of breeders not surviving past their first season [95,96]. In the laboratory, this species is highly susceptible, demonstrating 98% mortality across individuals sourced from four populations (*n* = 277; [19]). While some animals no doubt survive their first breeding season (potentially due to qualitative resistance or temperature-mediated cures) and might be expected to drive selection in subsequent breeding seasons for any traits they together possess, their genetic contribution to future generations is considerably weakened by the presence of successful offspring from the rest of their cohort that ultimately succumbed to infection.

Heterogeneity of tolerance at the subpopulation scale (e.g. between life stages, ages, sexes or size classes) should benefit the tolerant subpopulation, while potentially causing detriment via the increased force of infection on other less tolerant subpopulations. Such heterogeneity can dramatically shift underlying population structures (e.g. age structure and sex ratio) leading to increased population vulnerability, particularly in the context of other threatening processes [92]. For example, high Bd-associated mortality rates of adult alpine tree frogs (*Litoria verreauxii alpina*; figure 4) that return to the water to breed, severely truncated the age structure of the populations from maximum longevity of 7 years down to a single age cohort of first-time breeders [94–96]. As already described, many chytridiomycosis systems feature higher tolerance in some life-history stages (e.g. tolerant larval stages; figure 2*b*). Aquatic tadpoles with low resistance but high tolerance can act as a biotic reservoir maintaining the environmental zoospore pool [32,34]. However, against a background of cross-species transmission, the benefit of tolerant tadpoles may outweigh their detriment if they act as an insurance subpopulation that can ‘rescue’ the population as a source of new recruits in the event of high post-metamorphic mortality rates [97]. However, despite their high tolerance, sublethal developmental effects from infection in tadpoles could impose a range of ecological costs to populations including (i) delayed metamorphosis, (ii) competitive disadvantage in size/mass and (iii) lower overall recruitment [98–100].

By contrast, metamorphs and newly metamorphosed juvenile amphibians are generally the life stages most susceptible to chytridiomycosis across species [31]. They typically exhibit the highest infection loads and prevalence, as well as highest mortality rates [31,101–103]. This heightened vulnerability is likely due to exceptionally low resistance and tolerance at this life stage (figure 2*b*), potentially caused by several mechanisms: (i) resistance is likely compromised due to physical restructuring of the immune system at this time [104] and the expansion of keratinized epidermis [105]; (ii) tolerance is likely compromised due to resource limitation as animals stop feeding during mouth restructure [106], and tissues are reorganized [104]. As these animals metamorphose and emerge from the environmental pool, high exposure risk combined with low resistance and tolerance can potentially lead to high mortality and recruitment bottlenecks (figure 2*b*; [37]). In addition to increasing the risk of local extirpation particularly in combination with other threatening processes, such age-class bottlenecks also have the potential to greatly disrupt metapopulation dynamics via reducing the abundance of dispersing juveniles/subadults and lowering opportunities for site recolonization.

### (e) Despite mass mortalities, selection for tolerance and resistance is likely to be dilute in chytridiomycosis systems

Simple theory suggests that because tolerance increases the force of infection, tolerant hosts have a fitness advantage as infection prevalence increases, promoting selection for tolerance traits, and thereby increasing their frequency within a population over an evolutionary timeframe [107]. Thus, tolerance traits should go to fixation, leading to tolerance homogeneity and stable coevolutionary dynamics [3,108]. This is in direct contrast with the expected effects of resistance, where reduced pathogen fitness selects for counter-adaptations, leading to unstable antagonistic coevolutionary dynamics (the host–pathogen arms race; [3,9,109,110]). However, while borne-out in some empirical scenarios [107,111], there are several counter-arguments to the tolerance fixation hypothesis suggesting instead the evolutionary maintenance of heterogeneity, namely (i) tolerance involves resource allocation costs [1,3,13], (ii) hosts are exposed to diverse pathogens/environments and tolerance traits are to a degree pathogen- and condition-specific [108,112,113], (iii) the expression of tolerance mechanisms varies throughout the course of infection [113] and (iv) selection on tolerance mechanisms varies with age and life stage [114].

The proposed difference in the stability of evolutionary outcomes between tolerance and resistance was the major argument made by Venesky *et al*. [18] a decade ago, calling for preferential selection of amphibians with tolerance (versus resistance) to chytridiomycosis via *ex situ* captive breeding programmes. However, regardless of whether tolerance fixation and subsequent evolutionary stability occur empirically in this system, the argument is still somewhat disingenuous in the context of chytridiomycosis being a multi-host pathogen. That is, evolution of resistance in one host species within a community may not constrain the abundance or distribution of the pathogen sufficiently to generate strong counter-selection. Selection is thought to proceed faster within narrower niches [115,116] and therefore selection pressure within a multi-host system should necessarily be lower if only some member species of the community pool of hosts evolve resistance [117]. Indeed, evidence to date suggests few signs of counter-adaptations (either increased or attenuated virulence) between historical (5–13 years prior) and more contemporary Bd isolates from a site of Bd emergence in Panama from 2004 to 2007 [118]. Thus, we believe there is currently little reason to constrain *ex situ* management selection approaches to either tolerance or resistance.

We turn our attention now to potential mechanisms by which intrinsic tolerance and resistance could be selected for naturally, and the likely strength of such selection if it occurs. As we have already mentioned, many chytridiomycosis systems are characterized by having an inherently tolerant tadpole stage meaning that infection rarely results in mortality pre-metamorphic climax. As such, while adaptive responses to infection during the larval stage might involve trait variation (e.g. variable infection intensity), plastic sublethal responses (e.g. altered developmental rates) and delayed fitness effects, resulting evolutionary selection must be realized through mortality or differential reproductive fitness in peri- and post-metamorphic stages. While this is certainly possible, in many species, metamorphosis is characterized by high and uniform susceptibility to infection due to unavoidable physical and physiological restructuring [31,101–104]. In such cases where infection is also highly prevalent at metamorphosis, widespread disease-associated mortalities could generate substantial selection pressure via a recruitment bottleneck. Importantly, however, in these cases the strength of selection on either larval or peri-metamorphic tolerance and resistance mechanisms might be overwhelmed by selection on other traits. For example, if timing of metamorphosis coincides with seasonal peaks in the force of infection, a resulting selective sweep towards expedited or stalled larval development could alter species phenology. Thus, other potential autecological, behavioural or physiological attributes (as opposed to intrinsic resistance/tolerance) might be evolutionarily favoured (e.g. greater mass/size or rapid terrestrial transition at metamorphosis).

In adult amphibians, there are multiple factors potentially involved in determining individual infection outcomes and their impact on lifetime reproductive success (and hence evolutionary selection) that are largely unrelated to either intrinsic resistance or tolerance. These factors include variable exposure risk, suitability of environmental conditions for maintenance of infection and the timing of exposure relative to important life events (such as breeding). These factors are likely to dilute the strength of selection for both resistance and tolerance. Indeed, compounding features of chytridiomycosis systems, such as recruitment bottlenecks, have the potential to dramatically reduce genetic diversity through stochastic factors largely unrelated to resistance/tolerance. However, there are scenarios where these factors could select indirectly for tolerance and resistance. For example, where resolution of infection is environmentally mediated (e.g. exposure to elevated temperatures), those animals with increased tolerance and/or resistance might have improved chances of surviving long enough to experience such a cure, relative to their less tolerant/resistant conspecifics.

Similarly, increased tolerance and/or resistance, and specifically the long subclinical period of chytridiomycosis, can increase the likelihood of adult amphibians successfully breeding prior to succumbing to disease, meaning that their offspring are not subject to selection (figure 4; [94]). Additionally, such scenarios could favour evolution towards high reproductive investment, as per the terminal investment hypothesis [119,120]. In both cases, subclinical survival time is a factor for indirect selection on tolerance and/or resistance. Unfortunately, selection for long subclinical periods does not necessarily alter the ultimate mortality rate from infection in systems where environmental conditions do not fluctuate beneficially. In these cases, longer subclinical periods could be selected for without fundamentally improving the long-term prospects for the population. Although a more nuanced selective process might occur with second and subsequent breeding seasons, many amphibian species already demonstrate highly positively skewed age distributions (including from non-disease-associated mortality factors), meaning that such added selective effects are likely to be minimal (figure 4).

## 3. Conclusion and practical applications to management

As one of the key host defence mechanisms against pathogens, infection tolerance is pervasive within chytridiomycosis systems. Here, we demonstrated via two generic examples of tolerance (e.g. tolerant larval stages and the long subclinical period of post-metamorphic amphibians) that infection tolerance (i) has important implications for pathogen spread and maintenance, (ii) drives some species to decline, while ‘rescuing’ others and (iii) contributes to the dilution of natural selection for both tolerance and resistance.

In terms of practical applications for management, assisted selection for tolerance and resistance are expected to be valuable mitigation approaches for target species that are either functionally extinct in the wild, continue to decline, or have been rendered vulnerable to decline through the emergence of chytridiomycosis. Such artificial or assisted selection approaches should be able to overcome several of the intrinsic system constraints that likely result in lower pressure of natural selection in wild systems (e.g. adults dying post-breeding, environmentally mediated infection resolution, variation in exposure risk, blanket-mortalities of peri-metamorphic juveniles, and variable selection pressures in multi-host–pathogen communities).

Controlled laboratory exposure experiments could be performed in juvenile, subadult and adult amphibians, measuring variables such as proportion infected, pathogen growth rates and time to develop clinical signs. If these experiments are immediately followed by treatment via antifungals or heat, then the more tolerant and resistant individuals could be selected as founders for breeding. Markers for tolerance and resistance could also be identified via genome-wide assisted selection from host DNA. Biomarkers could then enable the acceleration of generational selection (selecting at the larval stage via DNA) and would reduce overall costs (fewer subsequent exposure experiments required), while simultaneously maintaining as broad a diversity of the genetic pool as possible by increasing the number of founders. Unfortunately, in many susceptible species, particularly those that have already experienced declines, there may be inadequate baseline genetic diversity from which to draw potential resistance/tolerance traits. Introducing such traits via genetic engineering (e.g. CRISPR-cas9 approaches) is an exciting future direction for species' conservation.

In this context, having traits associated with both improved resistance and tolerance from which to draw should broaden our repertoire of potential targets. With widespread pathogen endemicity (of BdGPL), and the common presence of an environmental zoospore pool, we expect few detrimental repercussions from increasing the tolerance of a target vulnerable species within a community of other hosts. However, in certain systems where a tolerant reservoir species or super spreader contributes disproportionately to maintaining pathogen persistence and force of infection, targeting that species through exclusion or reduction might be a more immediate approach.

## Data Availability

This article has no additional data.
